# A handy approximation for a mediated bioelectrocatalysis process, related to Michaelis-Menten equation

**DOI:** 10.1186/2193-1801-3-162

**Published:** 2014-03-27

**Authors:** Uriel Filobello-Nino, Hector Vazquez-Leal, Brahim Benhammouda, Luis Hernandez-Martinez, Yasir Khan, Victor Manuel Jimenez-Fernandez, Agustin Leobardo Herrera-May, Roberto Castaneda-Sheissa, Domitilo Pereyra-Diaz, Juan Cervantes-Perez, Jose Antonio Agustin Perez-Sesma, Sergio Francisco Hernandez-Machuca, Leticia Cuellar-Hernandez

**Affiliations:** Electronic Instrumentation Faculty, Universidad Veracruzana, Cto. Gonzalo Aguirre Beltran S/N, 91000 Xalapa, Mexico; Electronics Department, National Institute for Astrophysics, Optics and Electronics, Luis Enrique Erro 1, 72840 Sta. Maria Tonantzintla, Mexico; Higher Colleges of Technology, Abu Dhabi Men’s College, Abu Dhabi, United Arab Emirates; Department of Mathematics, Zhejiang University, 310027 Hangzhou, China; Micro and Nanotechnology Research Center, Universidad Veracruzana, Calzada Ruiz Cortines 455, 94292 Boca del Rio, Mexico

**Keywords:** Michaelis-Menten kinetics, Perturbation method, Reaction/diffusion equation, Mediated bioelectrocatalysis

## Abstract

In this article, Perturbation Method (PM) is employed to obtain a handy approximate solution to the steady state nonlinear reaction diffusion equation containing a nonlinear term related to Michaelis-Menten of the enzymatic reaction. Comparing graphics between the approximate and exact solutions, it will be shown that the PM method is quite efficient.

## Introduction

Michaelis-Menten equation is used to describe the kinetics of enzyme-catalyzed reactions for the case in which the concentration of substrate is grater than the concentration of enzyme. These reactions are important in biochemistry because the most of cell processes require enzymes to obtain a significant rate (Michaelis and Menten 1913; Murray 2002). Enzymes are large protein molecules, which act as remarkably catalyst to speed up chemical reactions in living beings. With this end, they do work on specific molecules, called substrates; without the presence of enzymes, the majority of chemical reactions that keep living things alive would be too slow to maintain life (Michaelis and Menten 1913).

As it was already mentioned, the aim of this study is to find a handing approximate solution which best describes a reaction diffusion process related to Michaelis-Menten kinetics. Several oxidoreductase reactions such as quinones and ferrocenes consist of electrode reactions which allow conjugating between redox enzyme reactions and electrode reactions. The redox compound-mediated and enzyme catalysed electrode process is called mediated bioelectrocatalysis (Thiagarajan et al. 2011). Among its applications in engineering it is utilized for biosensors, bioreactors, and biofuel cells. Therefore, it is important the search for accurate solutions for this equation. Unfortunately, solving nonlinear differential equations is not a trivial process.

The Perturbation Method (PM) is a well established method; it is among the pioneer techniques to approach various types of nonlinear problems. This procedure was originated by S. D. Poisson and extended by J. H. Poincare. Although the method appeared in the early 19th century, the application of a perturbation procedure to solve nonlinear differential equations was performed later on that century. The most significant efforts were focused on celestial mechanics, fluid mechanics, and aerodynamics (Chow 1995; Filobello-Nino et al. 2013; Holmes 1995).

In general, it is assumed that the differential equation to be solved can be expressed as the sum of two parts, one linear and the other nonlinear. The nonlinear part is considered as a small perturbation represented by a small parameter (the perturbation parameter). The assumption that the nonlinear part is small compared to the linear is considered as a disadvantage of the method. There are other modern alternatives to find approximate solutions to differential equations describing some nonlinear problems such as those based on: variational approaches (Assas 2007; He 2007; Kazemnia et al. 2008; Noorzad et al. 2008), Tanh method (Evans and Raslan 2005), exp-function (Mahmoudi et al. 2008; Xu 2007), Adomian’s decomposition method (Adomain 1988; Babolian and Biazar 2002; Chowdhury 2011; Jiao et al. 2001; Kooch and Abadyan 2011,2012; Vanani et al. 2011), parameter expansion (Zhang and Xu 2007), homotopy perturbation method (Beléndez et al. 2009; Biazar and Aminikhan 2009; Biazar and Ghazvini 2009; El-Shaed 2005; Fathizadeh et al. 2011; Faraz and Khan 2011; Feng et al. 2007; Fereidoon et al. 2010; Filobello-Nino et al. [Bibr CR15][Bibr CR16]; Ganji et al. 2008,2009; He [Bibr CR21][Bibr CR22][Bibr CR23][Bibr CR24][Bibr CR26]; Hossein 2011; Khan et al. 2011,2013; Madani et al. 2011; Mirmoradia et al. 2009; Noor and Mohyud-Din 2009; Sharma and Methi 2011; Thiagarajan et al. 2011; Vazquez-Leal et al. [Bibr CR46][Bibr CR47]), and homotopy analysis method (Hassana and El-Tawil 2011; Patel et al. 2012), among many others.

Although the PM method provides, in general, better results for small perturbation parameters *ε*<<1; we will see that our approximation, besides of being handy, has good accuracy even for relatively large values of the perturbation parameter.

The paper is organized as follows. First, we introduce the basic idea of the PM method. Second, we provide an application of the PM method solving the bioelectrocatalysis process already mentioned. Next, we discuss significant results obtained by applying the method. Finally, a brief conclusion is given.

## Basic idea of perturbation method

Let the differential equation of one dimensional nonlinear system be in the form 1

where we assume that *x* is a function of one variable *x*=*x*(*t*), *L*(*x*) is a linear operator which, in general, contains derivatives in terms of *t*, *N*(*x*) is a nonlinear operator, and *ε* is a small parameter.

Considering the nonlinear term in (1) to be a small perturbation and assuming that its solution can be written as a power series for the small parameter *ε*2

Substituting (2) into (1) and equating terms having identical powers of *ε*, we obtain a number of differential equations that can be integrated, recursively, to determine the unknown functions: *x*_0_(*t*),*x*_1_(*t*),*x*_2_(*t*)…

## Approximate solution for the nonlinear reaction/diffusion equation under study

The equation to solve is 3

where *k* and *α* denote positive reaction diffusion and saturation parameters, respectively, for the mentioned process; *y* is the mediator concentration and *x* the distance (Thiagarajan et al. 2011).

It is possible to find a handy solution for (3) by applying the PM method, and identifying terms 45

We use Newton’s binomial to transform (3) into the following approximate form 6

identifying *α* as the PM parameter (see (2)), we assume a solution for (6) in the form 7

Equating terms with identical powers of *α*, it can be solved for *y*_0_(*x*),*y*_1_(*x*),*y*_2_(*x*), …, and so on. Later on will be seen that a very good handy result is obtained by keeping just the first order approximation. 89

The solution for (8) that satisfies the boundary conditions is given by 10

where *A* and *B* are constants given by 1112

Substituting (10) into (9), we obtain 13

To solve (13), we employ the variation of parameters method (Chow 1995) which requires evaluating the following integrals 14

where  and  are the solutions to the homogeneous differential equation 15

*W* is the Wronskian of these two functions, given by 16

and *f*(*x*) is the right hand side of (13).

Substituting *f*(*x*) and (16) into (14), leads to 1718

Therefore, the solution for (13) is written, according to method of variation of parameters, as 19

applying boundary conditions *y*_1_(0)=0 and *y*_1_(1)=0 to (19) results 

By substituting (10) and (19) into (7) we obtain a first order approximation to the solution of (3), as it is shown 20

We consider, as a case study, the following values for parameters: *α*=0.1, *α*=1, and *α*=1.5 for *k*=0.1,1,5,10,20,50, and 100.

## Discussion

Nonlinear phenomena appear in such broad scientific fields like applied mathematics, physics, and engineering. Scientists in those disciplines face, constantly, with the task of finding solutions for nonlinear ordinary differential equations. As a matter of fact, the possibility of finding analytical solutions for those cases is very difficult and cumbersome.

**Figure 1:**
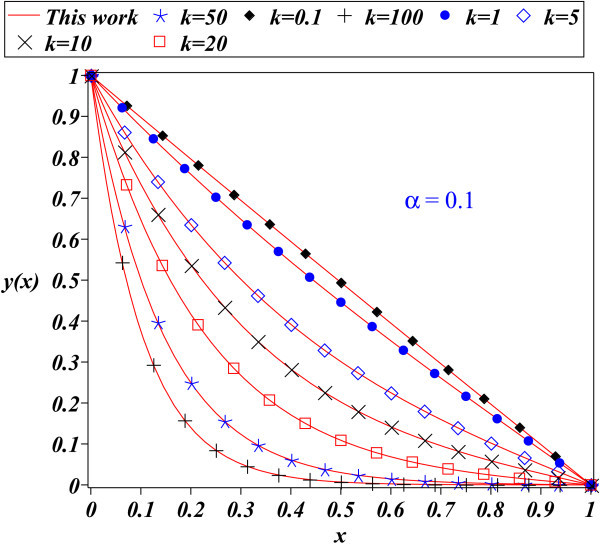
**Fourth order Runge Kutta numerical solution for (**3**) (symbols) and proposed solution (**20**) (solid line) for** ***α=0.1*** **.**

**Figure 2:**
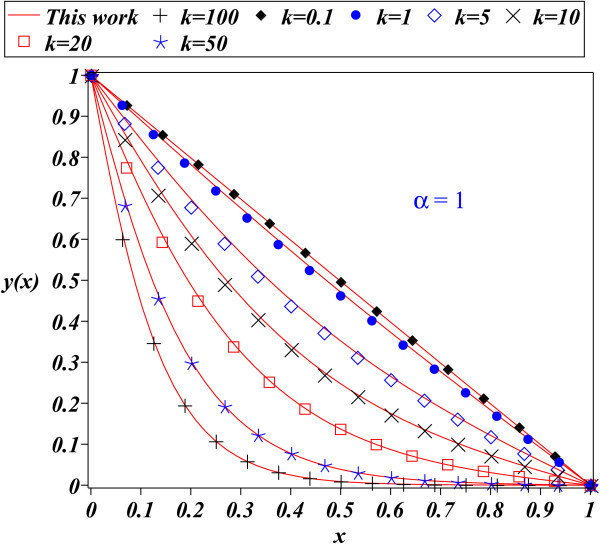
**Fourth order Runge Kutta numerical solution for (**3**) (symbols) and proposed solution (**20**) (solid line) for** ***α=1.0*** **.**

**Figure 3:**
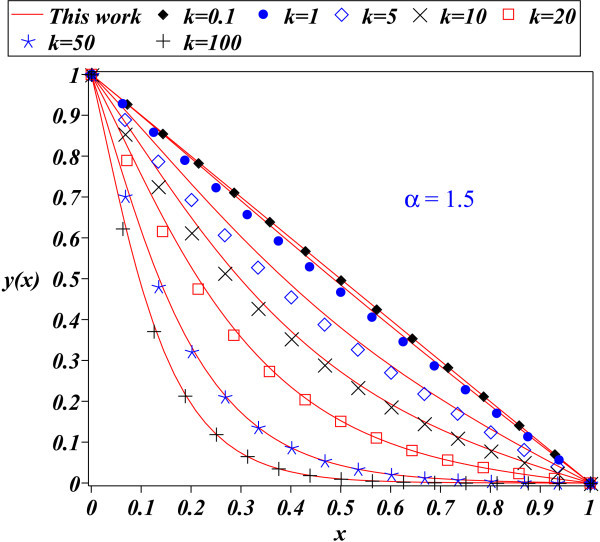
**Fourth order Runge Kutta numerical solution for (**3**) (symbols) and proposed solution (**20**) (solid line) for** ***α=1.5*** **.**

The fact that PM depends on a parameter, which is assumed to be small, suggests that the method is limited. In this work, the PM method has been applied to the problem of finding an approximate solution for the nonlinear differential equation which describes the time independent nonlinear reaction diffusion equation, corresponding to a nonlinear Michaelis-Menten kinetics scheme. This equation is relevant because its solution describes important applications such as biosensors, bioreactors, and biofuel cells, among others. Figures 1, 2, 3 show the comparison between approximation (20) for: *α*=0.1, *α*=1, and *α*=1.5 (*k*=0.1,1,5,10,20,50, and 100) to the fourth order Runge Kutta numerical solution. It can be noticed that figures are very similar for all cases, showing the accuracy of (20).

The PM method provides in general, better results for small perturbation parameters *ε*<<1 (see (1)) and when are included the most number of terms from (2). To be precise, *ε* is a parameter of smallness; measures how much larger is the contribution of linear term *L*(*x*) than *N*(*x*) in (1). Although Figure 1 for *α*=0.1 satisfies that condition, Figure 2 and Figure 3 show that (20) provides a good approximation as solution to (3); despite of the fact that perturbation parameters *α*=1 and *α*=1.5, cannot be considered small. Since that the transport and kinetics are quantified in terms of *k* and *α*, it is important that our solutions have good accuracy for a wide range of values for both parameters.

In (Thiagarajan et al. 2011), HPM method was employed to provide an approximate solution to (3). Although the solution reported has good accuracy, it is too long for practical applications. Unlike the above, (20) provides good accuracy, it is simple, and computationally more efficient.

Finally, our approximate solution (20) does not depend on any adjustment parameter, for which, it is in principle, a general expression for the exposed problem.

## Conclusion

An important task is to find an analytical expression that provides a good description of the solution for the nonlinear differential equations like (3). For instance, the time independent nonlinear reaction diffusion process, corresponding to a nonlinear Michaelis-Menten kinetic scheme is adequately described by (20). This work showed that some nonlinear problems can be adequately approximated employing the PM method, even for large values of the perturbation parameter; as it was done for the problem described by (3). The success of the method for this case has to be considered as an alternative to approach other nonlinear problems; this may lead to save time and resources employed using sophisticated and difficult methods. Figures 1 thru 3 show the accuracy of the proposed solutions.
